# Inhibition of fibrinolysis *in vitro* by supraphysiological doses of small-molecule factor XIa inhibitors

**DOI:** 10.1016/j.rpth.2026.106899

**Published:** 2026-08-04

**Authors:** Jelle Adelmeijer, Ton Lisman

**Affiliations:** Surgical Research Laboratory, Section of Hepatobiliary Surgery and Liver Transplantation, Department of Surgery, University Medical Center Groningen, University of Groningen, Groningen, The Netherlands

The coagulation system is intimately linked to the fibrinolytic system via various routes. One of these links concerns thrombin-mediated activation of thrombin-activatable fibrinolysis inhibitor (TAFI). Activated TAFI (TAFIa) cleaves C-terminal lysine and arginine residues from partially degraded fibrin [1]. This leads to attenuation of fibrinolysis, as these C-terminal lysine and arginine residues are binding sites for tissue-type plasminogen activator (tPA) and plasminogen. During clot formation, only small amounts of thrombin are required for fibrinogen-to-fibrin conversion, but ongoing thrombin formation eventually leads to activation of TAFI [2,3]. The “secondary burst” of thrombin required for TAFI activation is mediated by thrombin-induced activation of factor (F)XI [3]. A lack of secondary thrombin formation, as seen in FXI, FVIII, and FIX deficiency, leads to accelerated fibrinolysis due to reduced TAFI activation [[4], [5], [6]].

In plasma-based systems, deficiencies of FXI, FVIII, and FIX only lead to accelerated fibrinolysis when coagulation is initiated by a low concentration of tissue factor (TF) [6]. At higher TF concentrations, or at a low TF concentration in combination with a procoagulant trigger such as high-dose recombinant FVIIa [6,7], thrombin generation via the FVII-FX-FII pathway is sufficient for TAFI activation. In other words, only at low procoagulant triggers is thrombin-mediated activation of FXI required for sufficient thrombin generation to activate TAFI.

Anticoagulant drugs may not only inhibit coagulation but may also accelerate fibrinolysis by preventing secondary thrombin generation and thereby TAFI activation [[8], [9], [10], [11]]. The profibrinolytic effect of anticoagulant drugs may contribute to their antithrombotic effects as well as to bleeding.

Given the recent interest in FXI as an antithrombotic target [12], we explored the effects of 3 FXI(a)-targeting compounds in clinical development on fibrinolysis *in vitro*.

We studied lysis of a TF-induced clot by exogenous tPA by monitoring changes in turbidity during clot formation and subsequent lysis, as previously described [8]. Experiments were performed with both high and low TF triggers (Innovin diluted to final concentrations of 1000 or 100,000 times for “high” and “low” TF triggers, respectively). We added asundexian (MedChemExpress), milvexian (MedChemExpress), or abelacimab (MedChemExpress) to pooled normal plasma (a generous gift from Dr J.C.M. Meijers, Amsterdam University Medical Center, Amsterdam, The Netherlands) or FXI-deficient plasma (Stago). Clot lysis time (CLT) was defined as the time from the midpoint of the clear-to-maximum turbid transition, which characterizes clot formation, to the midpoint of the maximum turbid-to-clear transition, which represents clot lysis.

**Figure 1:**
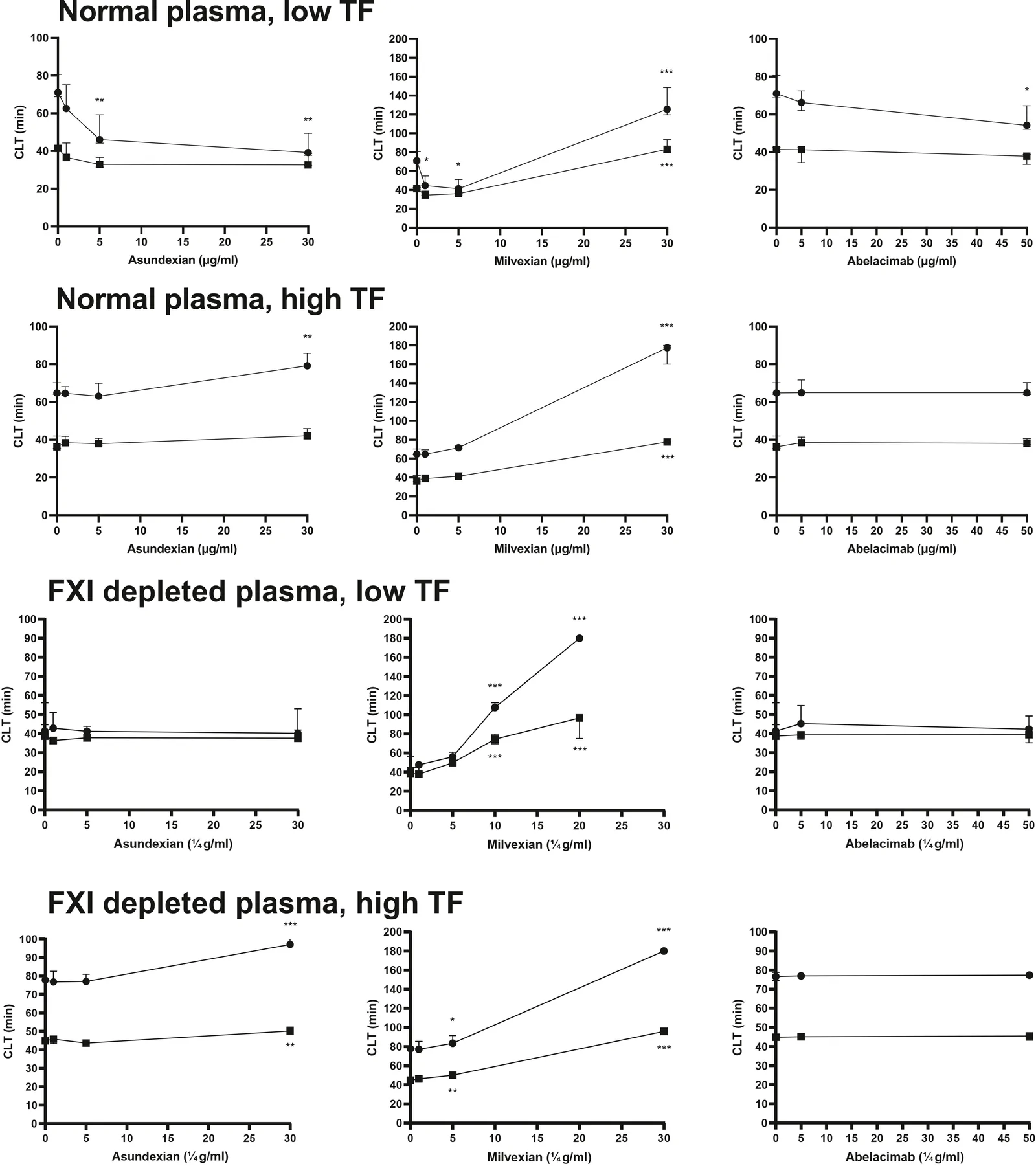
Pooled normal plasma or factor (F)XI-depleted plasma was supplemented with asundexian, milvexian, abelacimab (indicated concentrations are final concentrations in plasma), or vehicle, and clot lysis assays were performed at low or high tissue factor (TF) concentrations in the presence (squares) or absence (circles) of an inhibitor of activated thrombin-activatable fibrinolysis inhibitor. Clot lysis times (CLT) were calculated as previously described [8]. FXI-deficient plasma did not clot at milvexian 30 μg/mL, which is why intermediate concentrations were chosen. Data are medians with ranges of 3 independently performed experiments. ∗*P* < .05, ∗∗*P* < .01, ∗∗∗*P* < .001, all vs no drug added.

**Figure 2:**
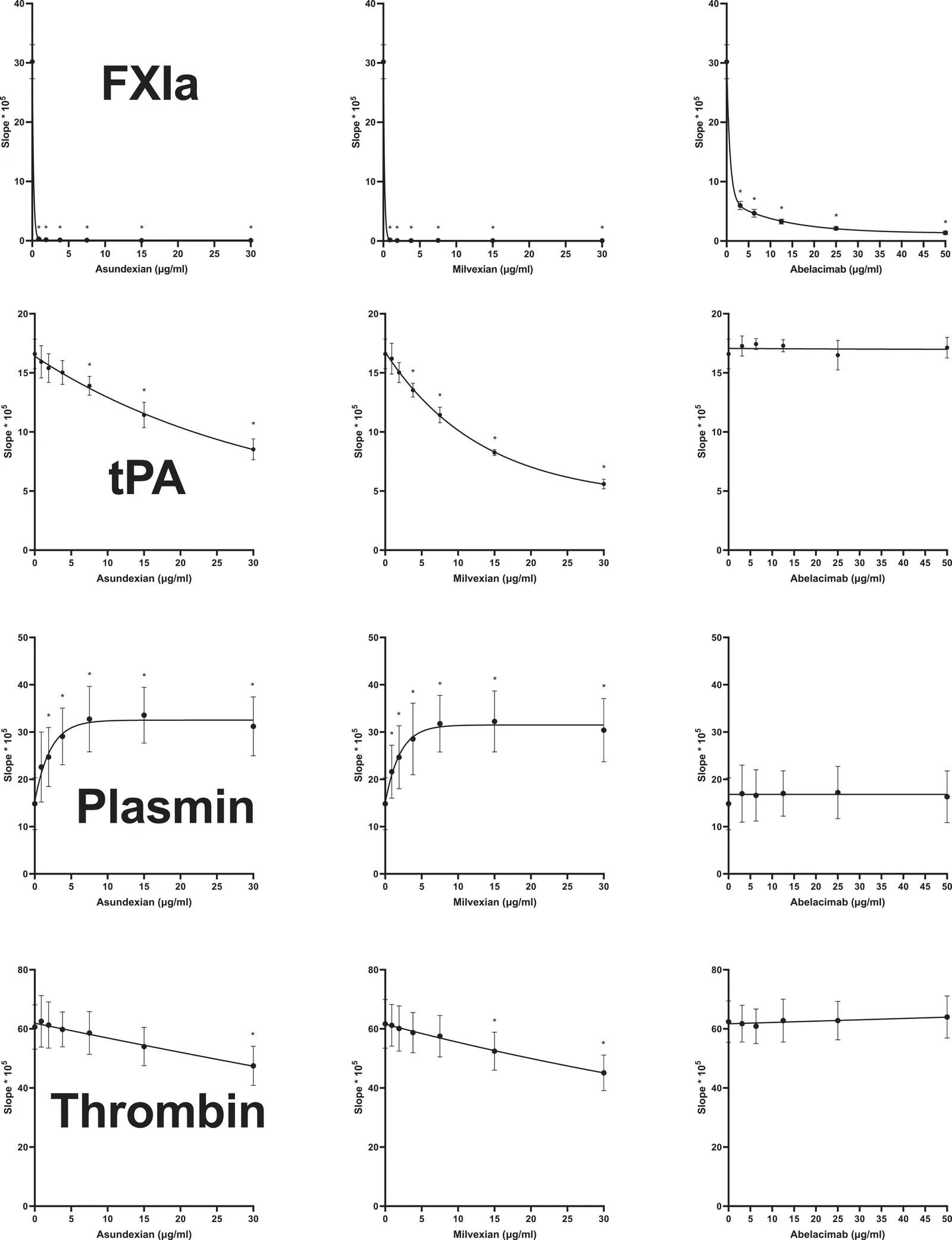
Factor (F)XIa, tissue-type plasminogen activator (tPA), plasmin, or thrombin was mixed with various concentrations of asundexian, milvexian, or abelacimab. After a 30-minute incubation, a chromogenic substrate was added, and absorption at 405 nm was measured over time. Shown are the slopes (optical density/s) of the regression lines for each drug concentration. Data are means ± SDs of 3 independently performed experiments. ∗*P* < .05 vs baseline values.

At a low TF trigger, the addition of asundexian, milvexian, and abelacimab at clinically relevant concentrations (±1 μg/mL for asundexian and milvexian, and 50 μg/mL for abelacimab) reduced CLT whereas CLT was unchanged at a high TF trigger at these concentrations (Figure 1). At supraphysiological concentrations of milvexian, CLT was substantially longer than in its absence, whereas after addition of supraphysiological concentrations of asundexian, CLTs were slightly but significantly higher than baseline only at a high TF trigger. In FXI-depleted plasma, asundexian and abelacimab had no effect on CLT at a low TF trigger, whereas at a high TF trigger, a supraphysiological dose of asundexian slightly but significantly increased CLT. In contrast, CLT increased dose-dependently when milvexian was added to FXI-depleted plasma. Supraphysiological concentrations of milvexian also increased CLT in the presence of a TAFIa inhibitor (DS-1040, 1 μg/mL, MedChemExpress) at both low and high TF triggers and in both normal and FXI-deficient plasma. Thus, milvexian and, to a lesser extent, asundexian at supraphysiological concentrations inhibited fibrinolysis in a FXI- and TAFI-independent manner. Remarkably, TAFIa inhibition decreased CLT at high milvexian concentrations in FXI-depleted plasma at low TF. Under these conditions, TAFI activation is not expected to occur; indeed, in the absence of milvexian, TAFIa inhibition does not affect CLT in FXI-depleted plasma at low TF (Figure 2). We have no clear explanation for why TAFIa inhibition decreases CLT at high milvexian concentrations under these conditions.

To explore mechanisms underlying the FXI- and TAFI-independent inhibition of fibrinolysis by asundexian and milvexian, we added plasmin (2.5 μg/mL, Abcam), tPA (56 ng/mL, Actilyse, Boehringer Ingelheim), FXIa (1 nM, Thermo Fisher Scientific), or thrombin (0.5 U/mL, Stago) to increasing concentrations of asundexian, milvexian, or abelacimab in a total volume of 80 μL buffer (20 mM Tris, 150 mM NaCl, 10 mM CaCl_2_, pH 7.5). After a 30-minute incubation at 37 °C, 40 μL of a chromogenic substrate (5-CHROM-03, 5-Diagnostics for plasmin; Biomed CS-05[88], Hyphen Biomed for tPA; S-2366, CoaChrom Diagnostica for FXIa; or 5-CHROM-81, 5-Diagnostics for thrombin) was added, and absorbance at 405 nm was measured over time at 37 °C. Slopes of the regression lines of the absorbance vs time curves were calculated and plotted.

Asundexian, milvexian, and abelacimab potently inhibited FXIa activity in a purified system (Figure 2). In addition, asundexian and milvexian inhibited tPA activity *in vitro* and paradoxically stimulated plasmin activity. Both drugs also inhibited thrombin activity. Abelacimab had no effect on tPA, plasmin, or thrombin activity. Since milvexian and asundexian are also potent inhibitors of kallikrein [13,14], and kallikrein has been implicated in fibrinolysis regulation [15], we assessed whether kallikrein inhibition contributes to the antifibrinolytic activity of high concentrations of milvexian and asundexian. Milvexian (30 μg/mL) increased CLT to a similar extent in pooled normal plasma, prekallikrein-depleted plasma, and plasma from an individual diagnosed with prekallikrein deficiency (a generous gift from Dr J.C.M. Meijers; data not shown), demonstrating that prekallikrein does not play a role in the antifibrinolytic effect of high concentrations of milvexian.

Thus, stimulation of fibrinolysis by clinically relevant doses of FXI(a) inhibitors may contribute to the antithrombotic effect of these drugs and support the use of antifibrinolytic agents, such as tranexamic acid, in managing bleeding complications associated with these drugs. Inhibition of fibrinolysis by supraphysiological concentrations of asundexian and milvexian appears related to the direct inhibition of tPA. Published data demonstrate that milvexian, but not asundexian, is an inhibitor of tPA [13,14]. However, these data were generated using compounds produced by the manufacturers of these drugs, whereas the data in the current work were generated using “biosimilars” produced and sold by a firm that uses in-house chemical synthesis to deliver high-quality life science reagents to researchers. Notably, biosimilars of asundexian and milvexian have been used in published research [[16], [17], [18], [19]]. Comparative studies assessing compounds made by companies producing these molecules as drugs and those producing them for use in research settings will be necessary to assess the relevance of the current findings. If confirmed, the current findings suggest that a cautious approach to dose escalations of asundexian and milvexian is imperative, as a hypofibrinolytic state induced by higher concentrations of these agents may lead to thrombotic events. Importantly, anecdotal evidence suggests that tPA deficiency or poor tPA release is associated with thrombotic disease [20,21], which may imply that tPA inhibition by supraphysiological doses of milvexian or asundexian may indeed be harmful. Nevertheless, the current experiments have only assessed the *in vitro* effects of FXI(a) inhibitors on fibrinolysis. Future studies should assess CLTs and markers of *in vivo* fibrinolysis activation, such as plasmin-antiplasmin complexes, in individuals receiving therapeutic and perhaps supratherapeutic doses of the FXI(a) inhibitor.
